# Therapeutic Time Window of Disease‐Modifying Therapy for Early Alzheimer's Disease in Japanese Individuals: Analysis Based on J‐ADNI Study

**DOI:** 10.1111/ggi.70698

**Published:** 2026-07-31

**Authors:** Saki Nakashima, Kenichiro Sato, Yoshiki Niimi, Ryoko Ihara, Kazushi Suzuki, Atsushi Iwata, Wataru Satake, Takeshi Iwatsubo, Japanese Alzheimer's Disease Neuroimaging Initiative

**Affiliations:** ^1^ Department of Neurology, Graduate School of Medicine The University of Tokyo Tokyo Japan; ^2^ Unit for Early and Exploratory Development The University of Tokyo Hospital Tokyo Japan; ^3^ Dementia Inclusion and Therapeutics The University of Tokyo Hospital Tokyo Japan; ^4^ Department of Neurology Tokyo Metropolitan Institute for Geriatrics and Gerontology Tokyo Japan; ^5^ Division of Neurology, Internal Medicine National Defense Medical College Saitama Japan; ^6^ National Center of Neurology and Psychiatry Tokyo Japan

**Keywords:** Alzheimer's disease, anti‐amyloid therapies, clinical dementia rating, disease‐modifying therapies, Japanese Alzheimer's disease neuroimaging initiative, therapeutic time window

## Abstract

**Background:**

Lecanemab and donanemab are recently approved disease‐modifying therapies (DMTs) for early Alzheimer's disease (AD), indicating amyloid positivity, with Mini‐Mental State Examination (MMSE) requirements. Prior analyses using North American population data suggested that baseline Clinical Dementia Rating–Global Score (CDR‐GS) and MMSE may define the “therapeutic time window,” but generalizability to Asian populations remains uncertain.

**Objective:**

To investigate the duration and predictors of the therapeutic time window, defined as the period until patients with early AD no longer meet eligibility criteria in Japanese patients.

**Methods:**

We retrospectively analyzed amyloid‐positive participants from Japanese Alzheimer's Disease Neuroimaging Initiative, classified as lecanemab‐eligible (MMSE 22–30, *n* = 129) or donanemab‐eligible (MMSE 20–28, *n* = 143). Kaplan–Meier survival was estimated over 24 months, and Cox proportional‐hazards models included age, sex, MMSE, CDR‐GS, and baseline diagnosis. Education years, *apolipoprotein‐E* ε4 (*APOE*‐ε4), and CDR‐Sum of Boxes (CDR‐SB) were tested individually.

**Results:**

At 12 and 24 months, survival probabilities for remaining eligible were 82% and 69% (MCI) versus 51% and 38% (AD) in the lecanemab group, and 92% and 81% (MCI) versus 69% and 52% (AD) in the donanemab group. Baseline CDR‐GS of 1 versus 0.5 predicted shorter eligibility for donanemab (HR = 2.50, 95% CI: 1.20–5.21), but not for lecanemab (HR = 0.48, 95% CI: 0.18–1.29). Each one‐point increase in MMSE above threshold was protective (HR = 0.67–0.68).

**Conclusions:**

Baseline CDR‐GS and MMSE strongly predict the therapeutic time window in Japanese patients, supporting cross‐population generalizability and contributing to the management of AD DMTs under resource constraints.

## Graphical abstract

In Japanese individuals with early Alzheimer's disease, baseline MMSE and CDR‐GS predicted the remaining therapeutic eligibility window for anti‐amyloid disease‐modifying therapies. These simple clinical measures may support patient triage, waiting‐list management, and timely treatment initiation under resource constraints.

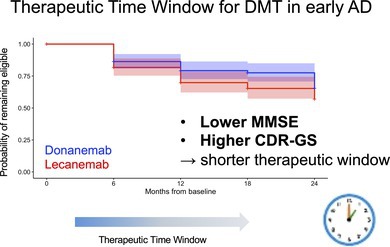

## 1 Introduction

Disease‐modifying therapies (DMTs) for early Alzheimer's disease (AD), such as lecanemab (Leqembi) [1] and donanemab (Kisunla) [2], have been approved in the United States, Japan, the United Kingdom, mainland China, Mexico, and several other regions, marking the beginning of a new era in AD treatment. In Japan, these agents are covered by the national health insurance system, offering new hope to patients with mild cognitive impairment (MCI) or mild AD dementia. The appropriate use recommendations (AUR) for lecanemab [3] specify that eligible patients are those with early AD who demonstrate evidence of amyloid pathology and have a Mini‐Mental State Examination (MMSE) score between 22 and 30. Although the AUR for donanemab describes an MMSE range of 20–30 [4], the pivotal clinical trial and current prescribing criteria for donanemab generally require patients to have amyloid positivity and an MMSE score between 20 and 28 [2, 5]. In Japan, the guidelines for both lecanemab and donanemab further stipulate that eligible patients have a Clinical Dementia Rating Global Score (CDR‐GS) of 0.5 or 1 [5, 6].

Given that AD is a chronic neurodegenerative condition characterized by gradual cognitive decline [7], the use of DMT is currently limited to individuals with early AD who meet these strict criteria, including a defined range for MMSE and CDR‐GS scores (for Japan). This may create a relatively narrow period during which patients remain eligible for receiving DMT, which can be called a “therapeutic time window,” similar to the concept applied in other conditions such as stroke [8].

Concerns have been raised regarding the readiness of healthcare systems to accommodate the provision of DMT [9, 10, 11, 12]. By June 2025, more than 8000 Japanese patients had received lecanemab infusion under health insurance during the first 18 months following approval [13]. Understanding the relationship between the therapeutic time window and patient wait times is critical to ensuring that eligible patients are not deprived of treatment opportunities. Investigating the therapeutic time window and its associated patient characteristics can also aid in patient management. Specifically, it can help predict the duration of eligibility, identify the timeframe within which AD DMTs could reasonably be initiated under hypothetical resource constraints, and make informed decisions on whether to retain patients on the waiting list or refer them to other facilities.

Our previous analysis using longitudinal observational data from the National Alzheimer's Coordinating Center (NACC) study and the Alzheimer's Disease Neuroimaging Initiative (ADNI) study [14] demonstrated that a higher baseline CDR‐GS and a lower baseline MMSE were associated with a shorter therapeutic window (pooled HR for CDR‐GS 1 vs. 0.5, 1.60; 95% CI, 1.26–2.04; per‐point increase in MMSE above the eligibility threshold, HR 0.66; 95% CI, 0.57–0.77); correspondingly, the estimated window length was over 12 months for most patients with baseline CDR‐GS 0.5 (approximately 75% remained eligible at 12 months), whereas about half of those with CDR‐GS 1 became ineligible within 1 year. However, these datasets predominantly consisted of approximately 90% White participants, limiting the generalizability of the findings to other populations, including Asia. To address this gap, we conducted a parallel analysis using data from Japanese ADNI (J‐ADNI) [15] to examine whether comparable patterns are observed in an Asian cohort.

## 2 Methods

### 2.1 Study Design and Participants

This is a retrospective study that utilized data from Japanese observational study databases. Participants included in the analysis were those considered eligible for lecanemab or donanemab at baseline for the respective time window assessment. The J‐ADNI study was launched in 2007 as a multicenter collaborative effort involving 38 clinical sites across Japan, imaging and biomarker researchers, industry researchers, and government agencies. The study conducted a detailed natural history follow‐up for 2–3 years on a total of 537 individuals, including 234 with MCI, 149 with mild AD, and 154 with cognitively normal elderly participants [15, 16]. A combined analysis of data from ADNI and J‐ADNI cohorts demonstrated that the trajectory of cognitive and clinical decline in individuals with “MCI due to AD,” was highly comparable between the Japanese and American population, supporting the notion that the pathophysiology and clinical course of early AD across ethnic groups [17]. As in the original ADNI protocol, the J‐ADNI enrollment criteria excluded neurological and psychiatric conditions that could potentially affect dementia progression, including Parkinson's disease, multiple cerebral infarctions, and major psychiatric disorders.

### 2.2 Eligibility Determination

Eligibility for lecanemab and donanemab in this study was determined based on clinical diagnosis and amyloid status, MMSE, and CDR‐GS. In pivotal U.S. clinical trials, eligibility for lecanemab and donanemab was primarily determined by clinical diagnosis and MMSE thresholds, and CDR‐GS was not applied as a strict inclusion criterion [1, 2]. However, AURs subsequently emphasized the importance of CDR‐GS, specifying a range of 0.5–1.0 to more precisely define the early AD population [3, 4]. In Japan, the Ministry of Health, Labour and Welfare (MHLW) went further by mandating CDR‐GS 0.5 or 1 in its official Optimal Use Guidelines for both lecanemab and donanemab [5, 6]. These developments underscore that CDR‐GS has become a central criterion in guiding treatment eligibility, particularly in clinical practice outside the original U.S. trial settings.

Amyloid positivity was defined according to previously published criteria [17] as either a visually positive read of the PiB PET scans [18], or a baseline cerebrospinal fluid (CSF) Amyloid β42 (Aβ42) concentration below 333 pg/mL. Standardized uptake value ratios (SUVRs) of PiB were calculated from J‐ADNI PET images using an established method, with a threshold of 1.48 [18]. Visual reads were adopted as the primary criteria for amyloid deposition, consistent with regulatory guidance from the U.S. Food and Drug Administration and Japanese guidelines, which endorse visual interpretation of amyloid PET images with 18F‐labeled tracers that demonstrate distribution patterns similar to PiB.

Participants were included if they were amyloid‐positive and diagnosed as MCI or mild AD. For lecanemab, eligibility was defined as an MMSE score of 22–30 and a CDR‐GS score of 0.5 or 1.0; for donanemab, eligibility was defined as an MMSE score of 20–28 and a CDR‐GS score of 0.5 or 1.0. These definitions were consistent with clinical trial inclusion criteria and current prescribing criteria [1, 2, 5, 6]. No additional criteria, such as the presence of more than four microhemorrhages or severe white matter hyperintensities, were applied, as these data were not consistently available.

At each follow‐up visit, participants were re‐evaluated for eligibility based on their MMSE or CDR‐GS. Because the therapeutic time window was intended to capture loss of eligibility due to clinical progression, the upper MMSE thresholds were applied only when defining the baseline drug‐specific cohorts. Loss of eligibility for lecanemab was defined by an MMSE score dropping below 22 or a CDR‐GS score exceeding 1, and for donanemab by an MMSE score dropping below 20 or a CDR‐GS score exceeding 1.

### 2.3 Statistical Analyses

We first conducted a survival analysis to determine the time (in months) until participants became ineligible for lecanemab or donanemab for the first time. A Cox‐proportional hazard analysis was used to evaluate the association of baseline characteristics with time to becoming ineligible: Baseline variables included age, sex (female or not), baseline MMSE scores, CDR‐GS (0.5 or 1.0), clinical diagnosis (MCI or AD), education years, apolipoprotein E (*APOE*)ε4 carrier status (with/without ε4 alleles[s]), and baseline CDR Sum of Boxes (CDR‐SB) scores. The hazard function was modeled as [14]: $\begin{array}{c} h\left(t|X\right)=h_{0}\left(t\right)\exp\left(\beta_{1}\;\mathrm{Age}+\beta_{2}\;\mathrm{Sex}+\beta_{3}\;\mathrm{CDR}1_{\mathrm{bl}}\right. \end{array}$ $\begin{array}{c} \left.+\beta_{4}\;\mathrm{MMS}E_{\mathrm{bl}}+\beta_{5}\;\text{diagnosis}+\beta_{6}\;X\right) \end{array}$, where *MMSE*_*bl*_ indicates the MMSE score at baseline, and *CDR1*_*bl*_ denotes whether the participant had a CDR‐GS of 1 at baseline. The variable *X* represents other covariates, including education years, *APOE* ε4 status, and baseline CDR‐SB. Hazard ratios (HRs) with 95% confidence intervals (CIs) were estimated for each variable. An HR was considered statistically significant if its 95% CI did not include 1. Because the first 1–2 years may represent the most clinically relevant period for evaluating the remaining therapeutic eligibility window, the model was also applied specifically to assess the risk of ineligibility within 2 years of baseline.

### 2.4 Ethics

This study was approved by the University of Tokyo Graduate School of Medicine institutional ethics committee (ID: 2025264NI). Informed consent was not required for this type of study because the study uses anonymized data only. AI‐assisted tools were used for language editing; authors take full responsibility for the content.

## 3 Results

### 3.1 Basic Demographics

Participant characteristics are summarized in Table 1. The lecanemab group included 129 participants, of whom 77 (59.7%) were diagnosed with MCI, and the donanemab group included 143 participants, of whom 64 (44.8%) were diagnosed with MCI. Of the 129 lecanemab‐eligible participants, 116 (89.9%) were also eligible for donanemab. These overlapping participants accounted for 81.1% (116/143) of the donanemab‐eligible population. Thirteen participants met only the lecanemab criteria, whereas 27 met only the donanemab criteria.

**TABLE 1:** Baseline characteristics of included participants in J‐ADNI cohort.

Characteristics	J‐ADNI	
	For examining lecanemab window	For examining donanemab window
No. of participants	129	143
Age (Median, IQR)	74.0 (68.8–77.8)	74.8 (69.7–78.5)
Sex: Female	64 (49.6%)	75 (52.4%)
Clinical diagnosis MCI (vs AD)	77 (59.7%)	64 (44.8%)
Amyloid positive (CSF or PET)	100.0%	
With *APOE‐ε4* allele	85 (65.9%)	92 (64.3%)
Education years (Mean, SD)	13.1 (2.8)	12.8 (2.8)
CDR‐GS 0.5 (vs 1)	117 (90.7%)	121 (84.6%)
CDR‐SB score (Median, IQR)	2.0 (1.0–3.0)	2.5 (1.5–3.5)
MMSE score (Mean, SD)	25.3 (2.2)	24.0 (2.3)

*Note:* Eligibility was defined as MMSE 22–30 for lecanemab and MMSE 20–28 for donanemab, with CDR‐GS 0.5 or 1.0 for both drugs.

Abbreviations: AD, Alzheimer's Disease; *APOE*, apolipoprotein E; CDR‐GS, Clinical Dementia Rating Global Score; CDR‐SB, Clinical Dementia Rating Sum of Boxes; CSF, cerebrospinal fluid; IQR, interquartile range; J‐ADNI, Japanese Alzheimer's Disease Neuroimaging Initiative; MCI, mild cognitive impairment; MMSE, Mini‐Mental State Examination; PET, positron emission tomography; SD, standard deviation.

The median age of the cohort was 74.0 years in the lecanemab group and 74.8 years in the donanemab group, approximately half of the participants were female, and all participants were confirmed to be amyloid‐positive. In addition, more than 60% of participants were *APOE* ε4 carriers, and most participants had a CDR‐GS of 0.5.

### 3.2 Survival Analysis on the Eligible Time Window

**FIGURE 1:**
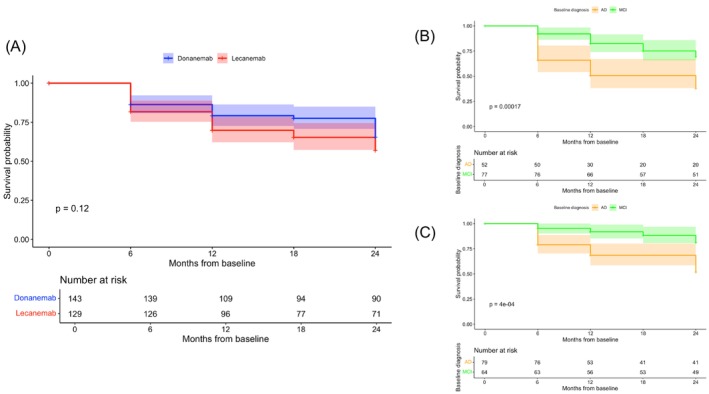
Kaplan–Meier survival curves until becoming ineligible. Kaplan–Meier survival curves illustrating the time until participants became no longer eligible are presented, focusing on the first 24‐month timeframe. Eligibility was defined as MMSE 22–30 for lecanemab and MMSE 20–28 for donanemab, with CDR‐GS 0.5 or 1.0 for both drugs. The figures are organized by drug type (A), baseline diagnosis in the lecanemab‐eligible cohort (B), and baseline diagnosis in the donanemab‐eligible cohort (C). AD, Alzheimer's Disease; MCI, mild cognitive impairment.

Kaplan–Meier survival curves illustrating the time until participants became no longer eligible are presented in Figure 1, focusing on the first 24‐month timeframe. The figures are organized by drug type (Figure 1A) and clinical diagnosis at baseline (AD vs. MCI, Figure 1B,C). Regardless of the drug type, individuals with a clinical diagnosis of AD at baseline had a significantly shorter eligible window period than those with a clinical diagnosis of MCI (*p* < 0.001, log‐rank test).

The estimated survival probabilities for remaining eligible were 69.8% at 12 months and 57.0% at 24 months in the lecanemab group and 79.2% at 12 months and 65.5% at 24 months in the donanemab group (Figure 1A). When stratified by baseline diagnosis, survival probabilities in the lecanemab group were 82.3/69.2% (12/24 months) for participants with MCI and 50.6/38.0% (12/24 months) for those with AD (Figure 1B). In the donanemab group, corresponding probabilities were 91.8/81.2% (12/24 months) for participants with MCI and 68.5/51.8% (12/24 months) for those with AD (Figure 1C).

### 3.3 Factors Associated With the Eligibility Time Window Length

**FIGURE 2:**
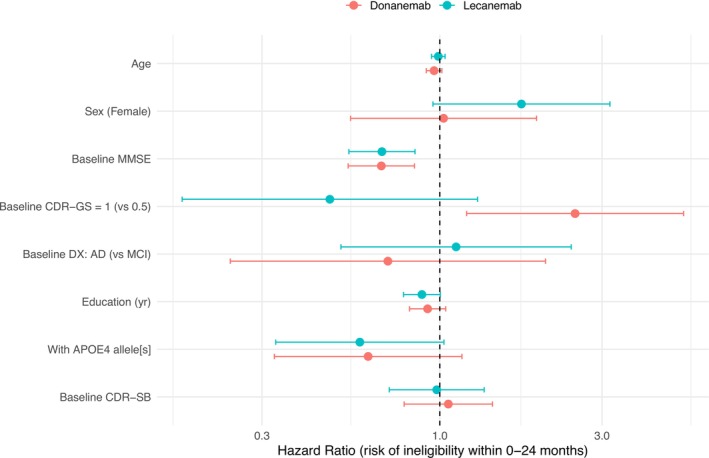
Cox‐Proportional Hazard Results. The results of the Cox‐proportional hazard analysis for events within 24 months from baseline are shown with the logarithm of HR. Age, sex, baseline MMSE, CDR‐GS, and baseline diagnosis were commonly included in the models, while education years, APOE4 allele status, and baseline CDR‐SB were entered one by one as additional covariates. Baseline MMSE was modeled as the number of points above the drug‐specific lower eligibility threshold. An HR was considered statistically significant if its 95% CI did not include 1. Better baseline cognitive performance predicted a longer eligibility window regardless of drug type. AD, Alzheimer's Disease; CDR‐GS, Clinical Dementia Rating Global Score; CI, confidence interval; HR, hazard ratio; MCI, mild cognitive impairment; MMSE, Mini‐Mental State Examination.

The results of the Cox‐proportional hazard analysis for events (becoming ineligible) within 24 months from baseline are shown in the forest plots (Figure 2). In drug‐specific analyses, a baseline CDR‐GS of 1 versus 0.5 was associated with a markedly higher risk of becoming ineligible within 24 months for donanemab (HR = 2.50, 95% CI: 1.20–5.21), whereas no significant association was observed for lecanemab (HR = 0.48, 95% CI: 0.18–1.29). Conversely, each one‐point increase in MMSE from the lower eligibility threshold was consistently associated with a lower risk of ineligibility (HR = 0.67, 95% CI: 0.54–0.84 for donanemab; HR = 0.68, 95% CI: 0.54–0.85 for lecanemab), indicating that better baseline cognitive performance predicted a longer eligibility window regardless of drug type.

## 4 Discussion

The guidelines for lecanemab and donanemab stipulate recommendations and requirements for patient eligibility, thereby necessitating consideration of the therapeutic time window. We previously analyzed NACC and ADNI datasets and found that higher CDR‐GS and lower MMSE at baseline were associated with a shorter therapeutic eligibility window (pooled HR 1.60 for CDR‐GS 1 vs. 0.5; HR 0.66 per one‐point increase in MMSE); approximately half of those with CDR‐GS 1 lost eligibility within 12 months [14]. As these datasets included more than 90% White participants, generalizability to Asian populations was uncertain. We therefore examined a Japanese cohort.

In our J‐ADNI results, MMSE remained consistently protective across agents (HR 0.67–0.68 per point), whereas the effect of CDR‐GS appeared stronger for donanemab (HR 2.50, 95% CI 1.20–5.21) and was not statistically significant for lecanemab. This difference in statistical significance should be interpreted with caution. Although the lecanemab‐ and donanemab‐eligible cohorts overlapped substantially, they were not fully nested because the MMSE ranges differed between the two definitions. The lecanemab‐eligible population was smaller and included fewer participants with baseline CDR‐GS of 1, which likely limited statistical power. Therefore, the present data do not allow firm conclusions regarding drug‐specific differences in the prognostic impact of CDR‐GS. Only a minority became ineligible within 6 months, and more than two‐thirds remained eligible at 12 months, suggesting a slower decline in eligibility than in the North American cohort. These differences may reflect healthcare system factors, but case‐mix likely contributes: Compared with ADNI, the Japanese cohort included slightly more women and a higher proportion with baseline CDR‐GS of 0.5, which alone could plausibly lengthen the therapeutic time window. Regarding sex composition, a resilience‐based explanation is plausible: Sex‐related differences in cognitive resilience, including advantages in verbal memory and compensatory strategies more frequently observed in women [19], may buffer the clinical impact of pathology, thereby prolonging the observed eligibility window in the Japanese cohort. This interpretation is supported by a recent tri‐cohort analysis integrating ADNI, J‐ADNI, and Australian Imaging Biomarkers and Lifestyle Study of Aging (AIBL), in which women exhibited earlier hippocampal involvement and greater white‐matter abnormality yet less longitudinal cognitive decline than men [20]. Conversely, other high‐impact syntheses summarize datasets in which women progress faster after MCI/AD diagnosis [21]. Considered together, these findings indicate that, although women are sometimes reported to exhibit faster pathological progression, sex differences in the rate of cognitive decline are not consistent across cohorts; in our Japanese cohort, women appeared to decline more gradually in cognition, consistent with the hypothesis that sex‐related cognitive resilience—such as advantages in verbal memory—may attenuate the clinical expression of underlying pathology.

However, in the Japanese cohort, the direction of effects was broadly replicated; while eligibility appeared to decline more gradually than in North American cohorts, the protective association of MMSE and the adverse association of higher CDR‐GS were consistently evident. When planning PET or CSF scheduling or initiating DMT administration, dementia specialists can use our results to roughly estimate the duration of the therapeutic time window, even if many patients at the diagnostic stage ultimately prove ineligible. Such information may support appropriate patient management for DMT in general, particularly in facilities facing delays in the care pathway. In this context, baseline MMSE and CDR‐GS—which are readily available in routine clinical practice—may serve as practical indicators for patient triage, prioritization, and waiting‐list management when access to diagnostic resources or infusion capacity is limited. Moreover, quantifying the expected duration of eligibility based on these baseline measures provides a practical framework for estimating how long patients may remain within the therapeutic window, thereby supporting healthcare system‐level planning and facilitating comparisons across populations. Because MMSE and CDR‐GS are themselves components of the eligibility criteria, these findings should be interpreted as quantifying how baseline position within the eligibility range relates to subsequent loss of eligibility, rather than as identifying independent etiological predictors.

Previous studies conducted in 2019 estimated the potential wait times for the initiation of DMT in European countries to be as long as 1 year [9, 10]. In contrast, a recent survey study in Japan, 2024–2025 reported that most specialist respondents experienced average wait times of 3 months or less from the initial consultation to the first infusion [22]. Our survival analyses demonstrated that only a small population of participants became ineligible within 6 months (Figure 1), suggesting that the current scheduling practices are generally feasible and acceptable in usual‐care scheduling. The clinical pathways established under Japan's universal health coverage, together with the country's abundant MRI capacity [23], likely contribute to this timely access. Nevertheless, maintaining this level of access as the eligible patient population grows will be challenging. The number of eligible patients in Japan is projected to increase to approximately 32 000 annually by 2031 [24], which could result in longer waiting times and underscore the clinical importance of considering the therapeutic time window.

There are several possible strategies to shorten this therapeutic time window. To confirm amyloid pathology, PET and CSF assessments are important; however, timely access to these modalities remains limited. The integration of blood‐based biomarkers (BBM) into prescreening may help mitigate these resource constraints by reducing dependence on PET and CSF, which are time‐consuming and invasive. In Japanese clinical practice, if reimbursed under health insurance as an adjunctive diagnostic tool, BBM assessment could be effectively performed by primary care physicians. For example, plasma Aβ42/Aβ40 ratios, where lower values are indicative of amyloid pathology, and phosphorylated tau markers such as p‐tau217, where higher levels reflect AD‐related changes [25, 26, 27], could be used to triage patients. In addition, identifying potential patients who are interested in DMT but lack access to accurate information is also critical. Web‐based surveys may represent a feasible approach for identifying and engaging such individuals. By better understanding the backgrounds and expectations of future participants, the design of upcoming online studies or surveys in the field of AD could be optimized [28].

Our study has several limitations. First, we did not consider additional exclusion criteria for determining DMT eligibility, such as the presence of multiple microhemorrhages or brain lesions consistent with vascular dementia [3]. Second, because of the observational nature of the study, in which data were collected primarily at 6 or 12‐month intervals [29, 30], the estimated eligibility range was strongly influenced by the structured visit schedule and may not accurately reflect the actual time course in real‐world clinical practice. Third, the present study was not designed to directly compare drug‐specific differences. Although the lecanemab‐ and donanemab‐eligible cohorts overlapped substantially, they were not fully nested because the MMSE ranges differed between the two definitions. The lecanemab‐eligible population was smaller and included fewer participants with baseline CDR‐GS of 1. Consequently, the number of ineligibility events in the lecanemab group was limited, which may have reduced statistical power to detect an independent effect of CDR‐GS. Therefore, our data do not allow firm conclusions regarding drug‐specific differences in the prognostic impact of CDR‐GS. Finally, the decision to initiate treatment for an individual patient requires careful consideration of potential risks and benefits, patient preference, and the availability of healthcare resources. As the disease progresses, the likelihood of further decline increases, favoring earlier treatment initiation; however, in more advanced stages, the risk–benefit balance may instead support withholding therapy. Therefore, the findings of this study should be interpreted as one element within a broader and more nuanced clinical decision‐making framework. Baseline CDR‐GS emerges as a strong predictor of the therapeutic time window for early AD DMTs also in the Asian population, supporting cross‐population generalizability, providing important information on patient prioritization and scheduling for AD DMTs under resource constraints.

## 5 Conclusion

We examined the duration of the therapeutic time window for DMT eligibility in early AD and demonstrated that a higher baseline CDR‐GS and lower MMSE were significant predictors of a shorter remaining time until ineligibility in the Japanese population. These findings support the cross‐population generalizability of previous results and provide important evidence to guide patient management for AD DMTs under resource‐constrained conditions.

## Funding

This work was supported by the Japan Agency for Medical Research and Development (24dk0207068, 25dk0207075) and the Japan Society for the Promotion of Science (JP24K10653, JP25K19014).

## Ethics Statement

This study was approved by the University of Tokyo Graduate School of Medicine institutional ethics committee (ID: 2025264NI).

## Consent

The authors have nothing to report.

## Conflicts of Interest

K.S. (Sato) is involved in a joint research project with the MetLife Foundation, and has received a research grant from Eli Lilly for collaborative research unrelated to the current manuscript. Y.N. is involved in collaborative researches with NIPRO Corporation, CANON Medical Systems Corporation, and Eli Lilly & Company, and had received consultancy/speaker fees from Eisai, and Eli Lilly. R.I. received advisory fees from Eisai, Eli Lilly, and MSD; consultant fee from Chugai; and honoraria for lectures from Eisai, Eli Lilly, Nihon Medi‐Physics, PDR Pharma, FUJIREBIO, Sysmex, and IQVIA. A.I. received research grants from Eisai, FUJIREBIO, Janssen pharma, Sysmex, Kobayashi Pharma, Eli Lilly, Fujifilm, SONY, Biogen, and Chugai/Roche; advisory fees from Eisai, FUJIREBIO, Eli Lilly, Roche, GSK, Otsuka, and Soundwave Innovation; honoraria for lectures from Eisai, Eli Lilly, Biogen, Chugai/Roche, HU frontier, FUJIREBIO, Kowa, Sysmex, Ono, Otsuka, Alnylam, Daiichi Sankyo, Tokio Marine & Nichido Fire Insurance, PDR pharma, IQVIA, Sumitomo Pharma, MSD, Janssen pharma, and Kyowa Kirin; patent assignment fee from FUJIREBIO; and is involved in postmarketing surveillance of lecanemab in Japan. T.I. had received consultancy/speaker fee from Biogen, Eisai, Eli‐Lilly, and Roche/Chugai. This manuscript has been prepared in a neutral and objective manner, and all disclosed financial relationships are not relevant to the content of this work. The remaining authors declare no conflicts of interest.

## Data Availability

The J‐ADNI study data is available from the website (https://humandbs.dbcls.jp/hum0043‐v1).
